# FlagT4G Vaccine Prevents Transplacental Transmission of Highly Virulent Classical Swine Fever Virus after Single Vaccination in Pregnant Sows

**DOI:** 10.3390/vaccines12080832

**Published:** 2024-07-23

**Authors:** Liani Coronado, Adriana Muñoz-Aguilera, Guillermo Cantero, Patricia Martínez, Mònica Alberch, Rosa Rosell, Douglas P. Gladue, Manuel V. Borca, Llilianne Ganges

**Affiliations:** 1 WOAH Reference Laboratory for Classical Swine Fever, IRTA-CReSA, 08193 Barcelona, Spain; liani.coronado@irta.cat (L.C.); adriana.munoz@irta.cat (A.M.-A.); guillermocanteroportillo@gmail.com (G.C.); patricia.martinez@irta.cat (P.M.); monica.alberch@uab.cat (M.A.); rosa.rosell@irta.cat (R.R.); 2 Unitat Mixta d’Investigació IRTA-UAB en Sanitat Animal, Centre de Recerca en Sanitat Animal (CReSA), Bellaterra, 08193 Barcelona, Spain; 3 IRTA, Programa de Sanitat Animal, Centre de Recerca en Sanitat Animal (CReSA), Bellaterra, 08193 Barcelona, Spain; 4 Subgerencia de Análisis y Diagnóstico, Instituto Colombiano Agropecuario (ICA), Bogotá 110221, Colombia; 5 Departament d’Agricultura, Ramadería, Pesca, Alimentació I Medi Natural i Rural (DAAM), 08007 Barcelona, Spain; 6 Plum Island Animal Disease Center, Agricultural Research Service, United States Department of Agriculture Greenport, Greenport, NY 11944, USA; douglas.gladue@usda.gov

**Keywords:** classical swine fever virus, transplacental transmission, FlagT4G vaccine, protection, neutralizing antibodies, immune response, vaccine efficacy

## Abstract

The transplacental transmission of CSFV and the resulting persistent congenital infection in newborn piglets have been abundantly discussed in pregnant sows suffering from virus infection. Importantly, the availability of safe commercial vaccines with proven efficacy to prevent the generation of congenital and postnatal persistent infections in pregnant sows are critical tools for controlling the disease in CSF endemic areas. Here, we demonstrate the high efficacy of a single dose of the recombinant FlagT4G vaccine to provide solid protection in pregnant sows against transplacental transmission of a highly virulent CSFV. Pregnant sows vaccinated with FlagT4G at 44 days of gestation elicited a strong CSFV-specific antibody response, with neutralizing antibody levels above those required for protection against CSFV. Importantly, after the challenge with a highly virulent CSFV, all foetuses from FlagT4G-vaccinated sows lacked CSF macroscopic lesions and showed a complete absence of the challenge virus in their internal organs at day 79 of gestation. Therefore, pregnant sows safely vaccinated with FlagT4G without affecting reproductive efficacy are efficaciously protected, along with their foetuses, against the infection and disease caused by a CSFV virulent field strain.

## 1. Introduction

Classical swine fever (CSF) is a highly contagious disease that affects swine, with a negative impact on pig production, animal health, and welfare. Therefore, it is a disease notifiable to the World Organization for Animal Health (WOAH) [1]. The etiological agent is classical swine fever virus (CSFV), which belongs to the *Pestivirus* genus within the *Flaviviridae* family [2,3]. 

CSF remains endemic in Asia, the Caribbean, and Central and South America [1]. In addition to its acute and lethal forms, in endemic regions, chronic, subclinical, and persistent disease forms are also prevalent, hindering control strategies. 

It is widely known that the CSFV transmission through the transplacental route, mainly during the second third of gestation, can result in persistently infected offspring [4,5,6,7,8]. Congenital persistent infection is one of the most important causes by which CSFV is perpetuated in the domestic pig population [8,9]. To control this type of infection, the vaccination of sows is essential, as is the use of rapid diagnosis and controls plans [1].

Live attenuated vaccines are widely used to control CSF disease in many countries, despite not having a marker character that differentiates vaccinated from infected animals (DIVA concept). Among these vaccine types, the C-strain is the most used and prevents 100% of clinical signs. However, field virus strains can persist even in areas where vaccination with this type of vaccine is mandatory [1].

In CSF-free countries, vaccination is usually prohibited, although it can, upon receiving an exception, be used in emergency situations. For this purpose, the use of marker vaccines with DIVA capability is preferred since they can reduce the impact of the disease on commercial trade [1]. The CSF live attenuated DIVA vaccine CP7_E2Alf, commercially called *Suvaxyn*, was licensed by the European Medicines Agency in 2014 [1,10]. CP7_E2Alf is a chimeric Pestivirus constructed on the backbone of the bovine viral diarrhoea virus (BVDV) in which the E2 gene was replaced by the CSFV Alfort/187 strain E2 gene. This vaccine is safe and effective compared to live attenuated vaccines, but with the DIVA added value. However, the protection conferred by this vaccine against transplacental transmission and highly virulent CSFV strains remains to be evaluated [10,11]. In addition, the antibodies conferred by this vaccine generate cross-reactivity with sera from animals infected with other pestiviruses when differential ELISAs based on the CSFV E^rns^ glycoprotein are used [12].

A new subunit vaccine strategy based on the E2 glycoprotein (Porvac^®^ Subunit Vaccine E2-CD154) has been shown to be effective in terms of clinical and virological protection as early as 7 days after a single vaccine dose [13]. This subunit vaccine is also DIVA compatible and confers protection from CSFV transplacental transmission after two vaccine doses [14].

Another live attenuated vaccine, FlagT4G, based on the CSFV Brescia strain, has been developed using a reverse genetic approach. A highly conserved CSFV-specific epitope of the E2 glycoprotein was eliminated, and 19 foreign amino acids were inserted into the E1 glycoprotein [15]. Recently, a differential diagnostic Flag-DIVA test has been developed, supporting the DIVA concept for this vaccine [16]. The Flag-DIVA test is a direct ELISA based on a dendrimeric peptide construct containing the highly conserved epitope of the E2 glycoprotein E2 that was eliminated in FlagT4G. The Flag-DIVA test detects anti-CSFV antibodies in infected animals but does not recognize the antibody response of FlagT4G-vaccinated animals [16]. Besides the DIVA concept, and given that FlagT4G induces effective immunity against CSFV challenge as early as 3 days after a single vaccination [17,18], this vaccine is a promising candidate for use against CSF [17].

The relevance of CSFV to be transmitted by transplacental route, as well as its ability to generate persistent infections, is well known; however, [6,7,19], it is worth highlighting the limited availability of scientific information that focuses on the efficacy of the live attenuated vaccines against CSFV, including the C-strain vaccine, in conferring protection in pregnant sows to avoid transplacental transmission [1,10,20].

Considering this background, the aim of the present work was to evaluate the capacity of the CSFV FlagT4G vaccine to confer protection in pregnant sows against transplacental transmission of a highly virulent CSFV strain. Coupled with this goal, we have elucidated the immunity that mediates the solid protection conferred, as well as the virological mechanisms behind the protection afforded by the vaccine.

## 2. Materials and Methods

### 2.1. Cells and Viruses

Viral production, virus isolation, titration, and neutralization assays were performed using porcine kidney cell line PK-15 (ATCC-CCL-33). This cell line was grown in Eagle’s minimum essential medium supplemented with 5% foetal bovine serum (FBS). Cell culture conditions were 37 °C with a 5% CO_2_ atmosphere in an incubator.

The CSFV FlagT4G vaccine virus [18] and the highly virulent Margarita (20) strain (genotype 1.4), were used in the in vivo experiments. A peroxidase-linked assay (PLA) was used for viral isolation and titration [21] following statistical methods [22]. The PLA test was also employed for monitoring viral replication.

The CSFV strain Alfort/187 and Diepholz1 were kindly provided by the CSFV EU Reference Laboratory (EURL), Hanover, Germany, and used for virus neutralization assays.

### 2.2. Experimental Design

Six pregnant Pestivirus-free sows (Landrace) 38 days into gestation were housed in the Animal Biosafety Level 3 (ABSL3) facility at IRTA-CReSA (Barcelona, Spain) and randomly allocated either to the vaccinated group (four sows, numbered 1–4) or the non-vaccinated group (two sows, numbered 5 and 6). After the acclimation period and following the protocol established in the WOAH Manual, at 44 days into gestation, the sows of the vaccine group were vaccinated intramuscularly with 10^5^ TCID_50_/mL of FlagT4G vaccine, while the control group remained unvaccinated [11]. At 65 days of gestation, 21 days post-vaccination (dpv), both groups (vaccine and non-vaccinated group) were challenged with 10^5^ TCID_50_ /mL of the highly virulent CSFV Margarita strain using the intramuscular route in the neck. After vaccination and CSFV challenge, a trained veterinarian recorded clinical signs daily in a blind manner, following a previously described methodology [14].

Serum, nasal, and rectal swab samples were collected on the day of vaccination and 10 dpv from vaccinated sows. Serum, nasal, and rectal swab samples were collected on the day of viral challenge and at 7- and 14-days post-challenge (dpc) from the six pregnant sows included in the present study (vaccinated and non-vaccinated animals). At the end of the trial (14 dpc), all the sows at 79 days of gestation (five weeks before farrowing) were euthanized. After necropsy, tissue samples from tonsils, mesenteric lymph nodes, and Peyer’s patches were collected. In addition, the foetuses from all sows were obtained, following procedures previously described to avoid fetal distress, and subjected to an exhaustive necropsy in which the presence of macroscopic lesions in different organs was evaluated. Serum and tissue samples (tonsil, spleen, and thymus) from foetuses were obtained. The experiment was approved by the Ethics Committee from the Generalitat of Catalonia under animal experimentation project number 10907, in accordance with Spanish and European regulations, on 3 September 2020.

### 2.3. Detection of Antibody Responses by ELISA and Virus Neutralization Tests

The detection of E2-specific antibodies was performed in serum samples using a commercial ELISA kit (IDEXX Laboratories, Liebfeld, Switzerland). The blocking percentage values were determined following manufacturer’s instructions: values below 30% were considered negative, between 30 and 40% were considered doubtful, and above 40% were considered positive. Additionally, serum samples were tested for neutralizing antibody titres against CSFV Margarita, FlagT4G, Alfort/187, and Diepholz strains using a neutralization peroxidase-linked assay (NPLA) [23]. The neutralization titres were expressed as the reciprocal dilution of serum that neutralized 100 TCID_50_ in 50% of the culture replicates. As previously established, neutralizing antibody titres of 1:35 were considered to have protective capacity against CSFV [24].

### 2.4. CSFV RNA Detection

Viral RNA was extracted from serum, nasal, and rectal swabs and tissue samples using the MagAttract 96 cador Pathogen Kit (Qiagen, Hilden, Germany) according to the manufacturer’s instructions. In all cases, RNA was extracted from an initial sample volume of 200 μL to obtain a final volume of 100 μL of RNA, which was stored at −80 °C. The supernatant of the tissue samples, previously ground in 900 µL of Eagle’s minimum essential medium, supplemented with 2% penicillin (10,000 U/mL) and streptomycin (10,000 U/mL) and centrifuged at 13,000 RPM for 10 min, was used for RNA extraction. For CSFV RNA detection, two RT-qPCR assays were used. One was used to detect the FlagT4G RNA vaccine, using the generic CSFV RT-qPCR test, which also detects all CSFV strains [25], and the other was used to detect the Margarita challenge RNA strain, which only amplifies the Margarita strain RNA [26]. Reactions were performed using the AgPath-ID™ One-Step RT-PCR Reagents (applied biosystems, Waltham, MA, USA). Samples were considered positive when the threshold cycle (Ct) values were equal to or less than 40 and negative when fluorescence was undetectable. Samples were also characterized as having high (Ct below 23), moderate (Ct between 23 and 28) and low (Ct between 29 and 40) viral RNA loads, as previously described [27]. To identify the CSFV RNAs of both viral strains (FlagT4G or Margarita) in the tonsils of vaccinated and non-vaccinated animals, the E2-gene fragment reported by Lowings et al. [28] was amplified by RT-PCR and sequencing using the BigDye terminator cycling technique and an ABI 3130xl genetic analyzer. Sequences were assembled using Contig BioEdit software (version 7.2) [29].

### 2.5. Determination of IFN-α Levels in Serum by ELISA Test

IFN-α concentration in serum was determined using a previously described in-house ELISA test [30,31]. Anti-IFN-α monoclonal antibodies (K9 and K17) and serial dilutions of recombinant IFN-α protein (PBL Biomedical Laboratories, Piscataway, NJ, USA) were employed as a standard in this assay. Serum samples from sows collected on the day of vaccination, 10 dpv, day of CSFV challenge (21 dpv), and 7 dpc, and serum samples collected from foetuses at necropsy were evaluated. The optical density of the standard was used to perform a regression curve and quantify the concentration of IFN-α in serum, with the results being expressed as units/mL.

### 2.6. CSFV Isolation in Tonsils Samples

Tonsils were used considering the relevance of this organ for CSFV replication [32].

Tonsil homogenate from samples collected from vaccinated and non-vaccinated sows, as well as from all the foetuses, were subjected to virus isolation. To this end, PK-15 cells were seeded in a 96-well plate and incubated at 37 °C and 5% CO_2_. After 24 h, 100 μL measures of 10- and 100-fold dilutions of each sample were added. After 72 h of incubation, the virus’ presence was detected using the PLA test [21].

## 3. Results

### 3.1. FlagT4G Vaccine Protects Pregnant Sows from Clinical Signs after Challenge with a Highly Virulent CSFV

**Figure 1:**
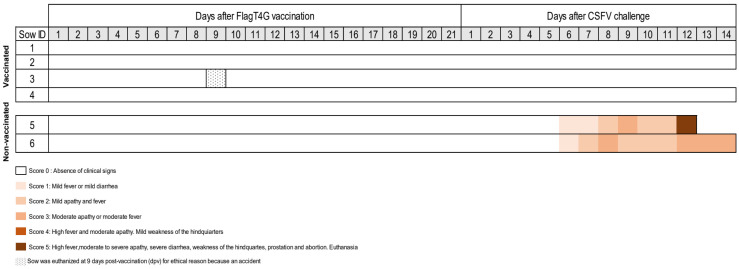
Clinical signs evaluated in pregnant sows after FlagT4G vaccination and CSFV challenge. The clinical signs were monitored daily during the trial. Different colours represent the severity of the clinical signs according to the legend. Sow number 3 was euthanized at 9 dpv, following animal welfare law, despite not showing clinical signs of CSF. Sows 5 and 6 were non-vaccinated, being the CSFV challenge control group.

After vaccination, no clinical signs were registered in vaccinated sows. Vaccinated sow number 3 had to be euthanized at 9 dpv in compliance with animal welfare regulations due to accidentally losing a hoof on one hind leg, even though the pen complied with the regulations for the animals’ habitat during the study (Figure 1). After CSFV challenge, the vaccinated sows remained healthy during the trial, with the absence of CSF clinical signs. In contrast, at six days post-challenge (dpc) onwards, non-vaccinated sows developed anorexia and mild–moderate apathy, progressing with diarrhoea and slight dyspnoea with nasal discharge (Figure 1). Remarkably, sow number 5 showed severe apathy, prostration, nasal discharge, and abortion at 12 dpc and was euthanized for ethical reasons (Figure 1). At 14 dpc (the end of the trial), all the sows in the study were euthanized at 79 days of gestation, five weeks before delivery. No lesions were found in any of the vaccinated sows after necropsy. Conversely, in both non-vaccinated infected animals, petechial haemorrhages in kidney, spleen, stomach, and intestine tissue were observed, which are all typical pathological findings typically observed in CSF.

### 3.2. FlagT4G Elicited Strong CSFV-Specific Antibody in Vaccinated Pregnant Sows

**Figure 2:**
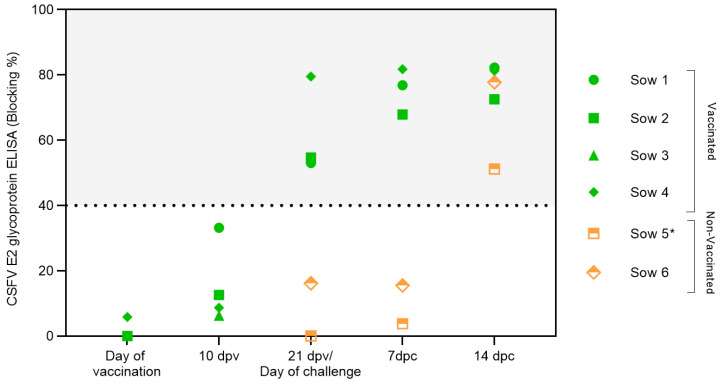
Antibody response generated in pregnant sows after FlagT4G vaccination and CSFV challenge. Sera samples from FlagT4G-vaccinated sows (represented in green symbols) and non-vaccinated sows (represented in orange symbols) were evaluated for the presence of antibodies against E2 using a commercial ELISA. The results are expressed in % blocking, and values above 40% (grey-shaded area) are considered positive. * Sample taken at 12 dpc.

Serum samples were analyzed to evaluate the induction of CSFV-specific antibodies after vaccination and CSFV challenge (Figure 2). Specific CSFV E2 glycoprotein antibody levels were detected in the vaccinated animals at 21 dpv. After challenge, an increase in the E2 antibody response was observed. The activation of the humoral response after vaccination, with a rapid increase after the viral challenge, was also detected by the serum neutralization assay (Table 1). High neutralizing antibody titres were recorded against a panel of CSFV strains from genotypes 1 and 2 at 21 dpv, reaching titres above 35 against the FlagT4G virus and Diepholz and Margarita strains. Considerable increases in the antibody titres at 7 and 14 dpc were detected in this experimental group (Table 1). Conversely, in the unvaccinated group, antibodies against the CSFV E2 glycoprotein were only recorded at the end of the study, i.e., at 12 dpc (sow number 5) or 14 dpc (sow number 6), (Figure 2). Regarding the neutralizing antibody, low titres were detected only in sow 5, which presented mild–moderate CSF clinical signs at 12 dpc. Sow number 6 remained negative for CSFV-specific antibodies during the trial (Table 1).

### 3.3. The CSFV-Induced Interferon Alpha-Exacerbated Response Is Prevented by FlagT4G Vaccine

**Figure 3:**
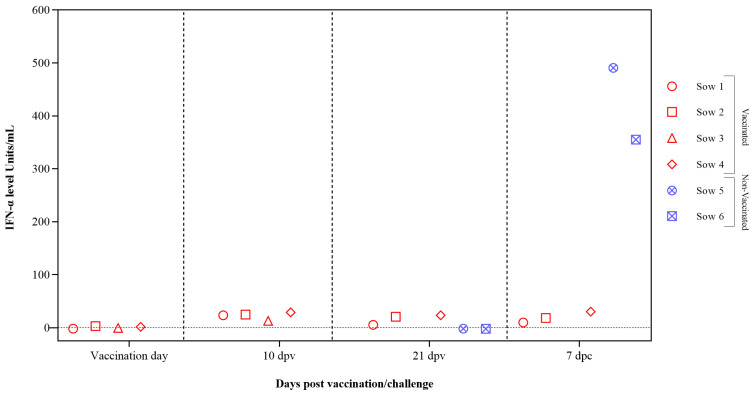
IFN-α response in sera from pregnant sows after FlagT4G vaccination and CSFV challenge. Sera samples from FlagT4G-vaccinated sows (represented in red symbols) and non-vaccinated sows (represented in blue symbols) were tested to analyze the response of the cytokine IFN-α. The results are expressed in units/mL.

Interferon alpha (IFN-α) concentrations in sera samples were evaluated after vaccination and CSFV challenge using the previously validated in-house ELISA test. Low or undetectable IFN-α levels were registered in vaccinated sows at 10 dpv with values below 30 units/mL at 7 dpc. In the case of the two non-vaccinated sows, high levels of IFN-α were detected at 7 dpc, reaching values of 300 and 500 units/mL (Figure 3).

### 3.4. FlagT4G Vaccine Avoids Systemic and Tissue Replication of the Highly Virulent Challenge Virus

**Figure 4:**
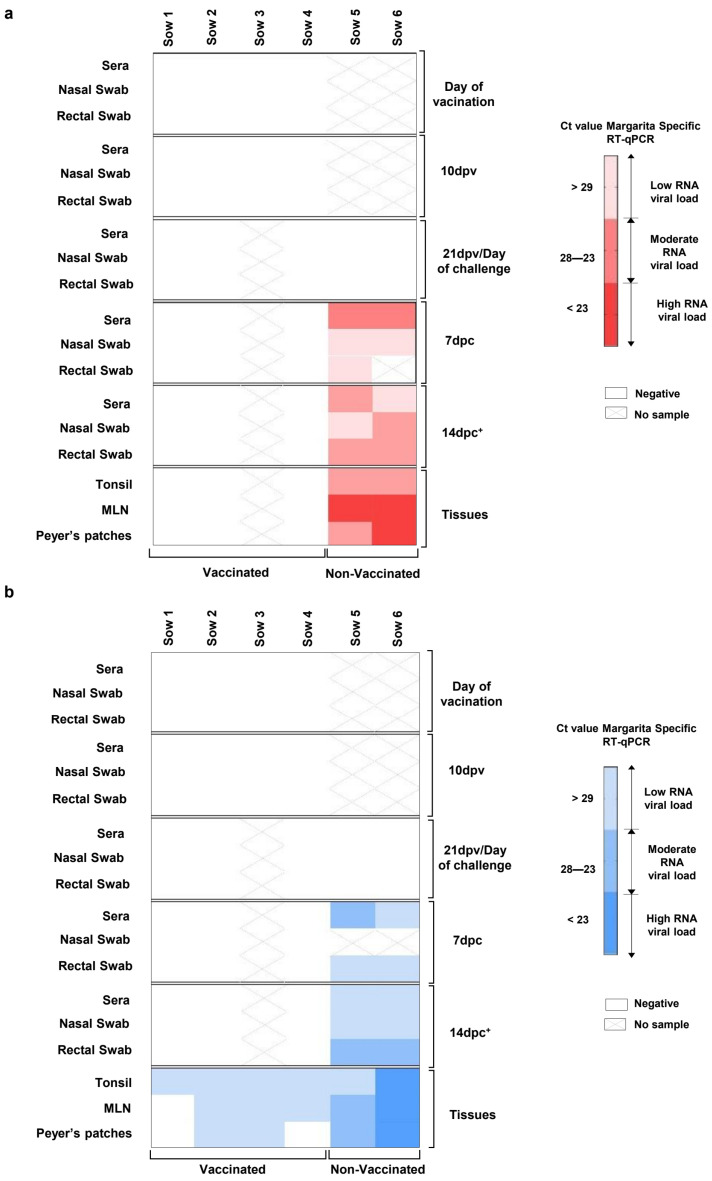
CSFV RNA detection in pregnant sows at different time points after vaccination and CSFV challenge in samples and tissues. The sample were analyzed by (**a**) the specific CSFV Margarita strain RT-qPCR test, represented in red, and by (**b**) the generic RT-qPCR test, represented in blue. The RNA load, according to the Ct value, is represented as low, moderate, or high according to the intensity of the red and blue colours in the scale for (**a**) and (**b**), respectively. Ct values above 40 were considered negative (represented by X). * The samples from sow 5 were taken at 12 dpc. MLN (mesenteric lymph node).

The CSFV Margarita strain RNA could not be detected by the CSFV Margarita specific RT-qPCR assay in any of the samples analyzed from vaccinated sows during the trial either after vaccination or after viral challenge (Figure 4A). Notably, the absence of FlagT4G vaccine RNA was found by generic CSFV RT-qPCR in all the serum, nasal and rectal swabs collected from vaccinated animals after vaccination and challenge (Figure 4B). In the case of the tonsil samples, all were positive according to the generic CSFV RT-qPCR test, with Ct values corresponding to low viral RNA load (Ct from 29.67 to 33.05). The mesenteric lymph nodes were also positive according to the generic CSFV RT-qPCR test in three out of four vaccinated sows, showing Ct values corresponding to low viral RNA load (Ct values from 32.21 to 34.73). For Peyer’s patches samples, only two out of four vaccinated animals were positive, as with Ct values corresponding to low viral RNA loads (Ct 32.78 and 33.49) (Figure 4B). 

In the case of samples coming from challenged–unvaccinated sows, CSFV RNA was detected from 7 dpc in all the serum, nasal, and rectal swabs tested by the two RT-qPCR assays used (Figure 4A,B). At this time, the Ct values detected corresponded with low-to-moderate viral RNA load. Subsequently, at 14 dpc, all serum and other swabs collected from these animals were positive by both RT-qPCR tests, with Ct values that corresponded to moderate-to-high viral RNA loads (Ct below 23) (Figure 4A,B). Likewise, tonsils, mesenteric lymph nodes, and Peyer’s patches from non-vaccinated–challenged animals were also positive using the two RT-qPCR tests, with Ct values corresponding to high RNA loads (Figure 4A,B). Tonsil samples that were positive using RT-qPCR tests with Ct values below 32 were also positive using the virus isolation test. The CSFV E2-gene fragment sequence obtained from the tonsils in non-vaccinated animals corresponding to the Margarita strain (AJ704817) was used as the inoculum. Conversely, in the tonsils from vaccinated sows, only the FlagT4G vaccine virus was identified and isolated in the animals numbered 1 and 3. 

### 3.5. FlagT4G Vaccine Afforded Total Protection from CSFV Highly Virulent Challenge in the Foetuses from Vaccinated Sows

**Figure 5:**
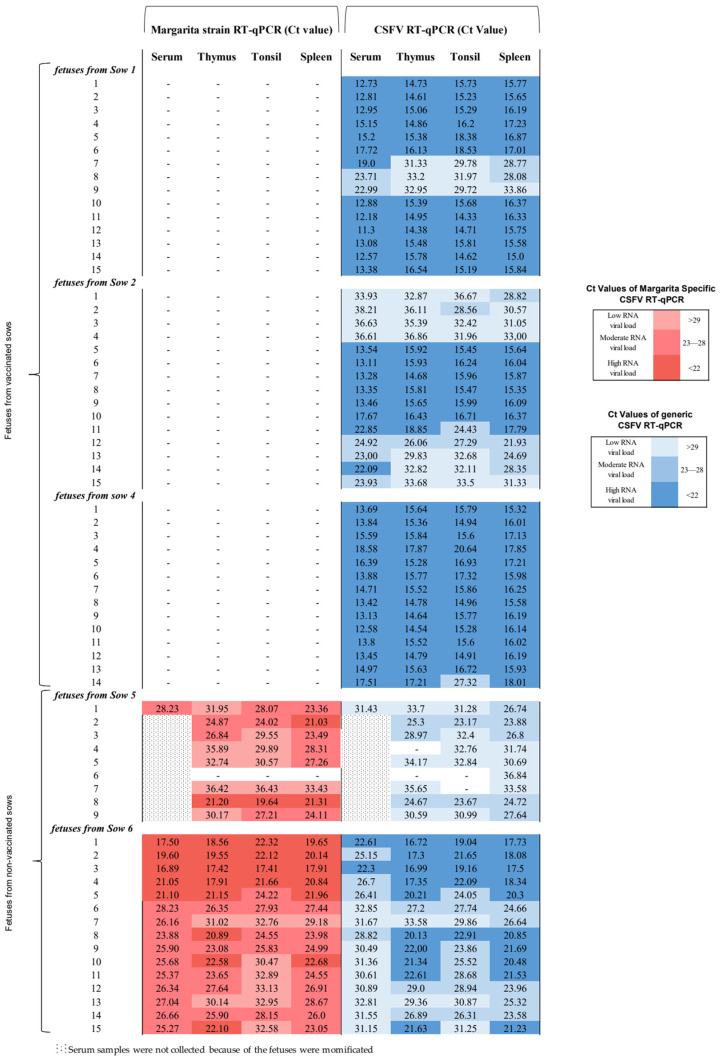
CSFV RNA detection by the RT-qPCR test in foetuses from vaccinated and non-vaccinated sows after CSFV challenge. Samples from foetuses were evaluated by the specific CSFV Margarita strain and the generic CSFV RT-qPCR assays. Results are shown in a colourimetric scale representing the range of values for the detection of both CSFVs: low for Ct values of 29 or above, moderate for Ct between 28 and 23, and high for values under 22. The results obtained are represented in red and blue for the specific CSFV Margarita and generic CSFV tests, respectively. The symbol “−” represents a negative result. In litter number 5, only one serum sample could be evaluated since the other foetuses in the litter were mummified after CSFV infection.

At 79 days of gestation, the foetuses from vaccinated sows were homogeneous in size, without macroscopic lesions or evidence of mummifications. Notably, a lack of CSFV RNA detection was found in all the serum, thymus, tonsil, and spleen samples collected in foetuses from vaccinated sows tested by the CSFV Margarita specific RT-qPCR assay (Figure 5). Nevertheless, in litters from non-vaccinated sows, a high number of mummified foetuses were found. In sow number 5, eight out of nine foetuses were mummified, and one was of small size in comparison to foetuses from vaccinated sows. In the case of sow number 6, the sow carried 15 foetuses of irregular size and with a significant number of lesions such as haemorrhaging on the skin, intestine, kidneys, and spleen. Considering that eight out of nine foetuses were mummified in litter number 5, only a serum sample could be collected. When these samples were evaluated by the CSFV Margarita-specific RT-qPCR, sera, thymus, tonsils, and spleen samples collected from foetuses in both non-vaccinated sows were positive after CSFV challenge. The Ct values registered correlated with a moderate-to-high viral RNA load (Ct between 17 and 28) (Figure 5). These results were also confirmed by the CSFV isolation test. Tonsils samples (six in total) from foetuses in litters 5 and 6, with Ct values above 32, were negative using the virus isolation test.

### 3.6. FlagT4G Vaccine Colonizes the Foetuses from Vaccinated Sows with a High CSFV RNA Load at Systemic and Tissue Levels

Notably, after using the generic CSFV RT-qPCR test for serum, thymus, tonsil, and spleen samples of foetuses from vaccinated and challenge sows (litters 1, 2 and 4), the samples were positive, with Ct values that correlated mostly with high CSFV RNA loads (Figure 5). In the case of litter from sow number 3 (euthanized due to an accident at 9 dpv), all serum samples were negative according to the generic CSFV RT-qPCR. Two thymus, three tonsil, and five spleen samples were positive with Ct values above 36, and one spleen was positive with a Ct value of 21. These results were confirmed by the CSFV isolation test. To identify the CSFV RNA in the tonsil from foetuses from vaccinated sows, the E2-gene fragment was once again amplified by RT-PCR and sequencing. Only the FlagT4G vaccine virus was identified in this experimental group.

### 3.7. CSFV Antibody and IFN-α Responses Were Not Found in the Foetuses from Vaccinated Sows

**Figure 6:**
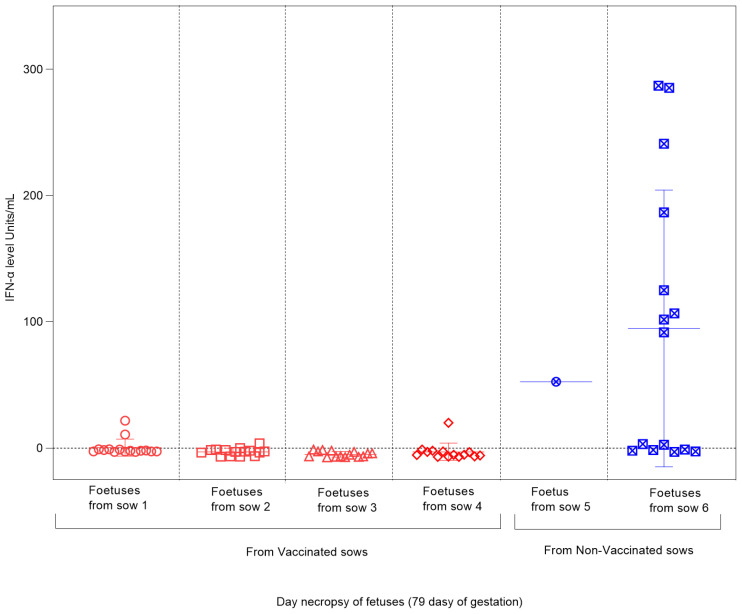
IFN-α level in foetuses from vaccinated and non-vaccinated sows after CSFV challenge. Sera samples of foetuses from vaccinated sows (represented by red symbols) and non-vaccinated sows (represented by blue symbols) were tested to analyze the response of the cytokine IFN-α. The results are expressed in units/mL. In litter number 5, only one serum sample could be evaluated since the other foetuses in the litter were mummified after CSFV infection.

The absence of CSFV-E2 glycoprotein-specific antibodies was found in all the foetuses during the study of both experimental groups. Regarding the Type I interferon response, measured by the IFN-α concentration in the sera, notably high levels were detected in the foetuses from non-vaccinated sows after infection with the CSFV Margarita strain, reaching values between 100 and 300 units/mL (Figure 6). For foetuses from vaccinated–challenged sows, negative results were obtained in most of the analyzed sera, with only two foetuses (one from sow 1 and one from sow 4) showing values below 30 units/mL (Figure 6).

## 4. Discussion

CSF congenital persistent infection and the CSFV capacity for transplacental transmission have been described for several decades; however, the pathogenesis and mechanisms of these aspects remain to be elucidated. Available information is limited and has not been updated [6,8,9,19]

Considering the widely known ability of CSFV to be transmitted via the transplacental route, the use of vaccines with proven efficacy in pregnant sows to prevent the generation of congenital and postnatal persistent infections, responsible for maintaining CSFV’s prevalence in endemic areas [8], is also a challenge today.

In the present study, the absence of macroscopic lesions, as well as the absence of the detection of the highly virulent challenge virus in all the samples analyzed in the foetuses from vaccinated sows, was founded. Notably, no evidence of CSFV specific antibodies was detected in these foetuses. In direct contrast—and in agreement with previous studies—foetuses from unvaccinated animals showed macroscopic lesions, with the detection of the high viral load of the challenge virus [5,14]. 

The FlagT4G-vaccinated pregnant sows at 44 days of gestation elicited strong CSFV-specific antibody responses, with neutralizing antibody levels above those required for protection against CSFV [24]. These neutralizing antibodies were effective against CSFV strains from genotypes 1 and 2. The activation of the humoral response after challenge was boosted, with a rapid increase after the highly virulent CSFV challenge in vaccinated sows. This suggests the presence of memory B cells activated by the FlagT4G vaccine with the capacity to enhance the reaction of the immune system to CSFV in vaccinated animals [1,17]. The high CSFV-neutralizing antibody levels induced upon vaccination and challenge correlated well with the strong protection observed in FlagT4G-vaccinated animals. These findings also correlated with the absence of clinical signs and absence of the detection of the highly virulent CSFV Margarita challenge strain. Interestingly, the CSFV-induced IFN-α exacerbated response was prevented by FlagT4G vaccine after viral challenge in the pregnant sows. This response correlated well with the clinical and virological protection observed. IFN-α is of great relevance in the innate immune response against viruses [33]. For CSFV, elevated levels of IFN-α generated rapidly and transiently after infection with highly virulent strains are related to disease severity after infection [27]. On the contrary, the CSFV challenged non-vaccinated sows showed abundant clinical signs, viremia, and pyrexia, and the highly virulent CSFV Margarita challenge strain could be detected in all analyzed samples coming from these animals, including prostration and abortion [5,14].

Neutralizing antibodies were only detected in low titres at 14 dpc (the end of this study) in one unvaccinated sow that developed moderate–severe CSF clinical signs. The IFN-α levels in the sera of unvaccinated sows were very high, supporting the role of the disproportionate IFN-α response in the generation of severe CSF disease [27]. 

As previously explained, in the present study, a single FlagT4G vaccine dose induced strong protection against the highly virulent challenge strain that is observed in 100% of the foetus samples collected at five weeks before delivery. This indicates that the effective immune response generated in sows after vaccination conferred protection against transplacental transmission. Notably, efficient transplacental transmission was detected in sows infected with both high- or moderate-virulence CSFV strains before the onset of antibody response, which started between 14 and 21 days post infection [5]. Previous studies also have shown that the tonsil is considered a target for CSFV replication, including vaccine viruses such as C-strain, which can persist in this tissue for more than 30 days after vaccination [34]. It is conceivable that, in addition to the tonsil, the CSF vaccine viruses can also be spread very quickly after vaccination to different organs, including through passage through the transplacental route. Unfortunately, there is limited availability of scientific information addressing the tropism of live attenuated CSFV vaccines and how long the vaccine viruses are detectable in samples from pregnant sows and foetuses after vaccination at different time points before birth.

As is well known, the placentas of sows is epitheliochorial. This prevents the transfer of maternal serum immunoglobulins to the foetus; hence, piglets acquire passive immunity via colostrum at birth [35]. The fact that the FlagT4G vaccine colonizes the foetuses with a high replication rate at the systemic and tissue levels, before the establishment of immunity in sows, supports the fact that in addition to the efficient pregnant sow’s immune response, another mechanism such as the superinfection exclusion (SIE) phenomenon is taking place. An increase in the IFN-α levels detected in foetuses after infection with high- or low-virulence CSFV strains has been reported [5]. Thus, the absence of IFN-α response in foetuses from vaccinated sows supports the strong virological protection conferred against the high-virulence strain and the possible implication of the SIE phenomenon.

The SIE is a natural phenomenon that occurs when a currently infected cell is resistant to secondary infection by the same or another closely related virus [36,37,38]. SIE has been applied in the treatment and prevention of viral infections [39]. It is a widely accepted practice for the cross-protection of crops. Also, it has been used for liver transplantation in patients infected by the hepatitis C virus, considering that SIE would prevent the reinfection of the transplanted organs [40,41]. It is worth highlighting the fact that like CSFV, many of the viruses that can generate SIE are single-stranded, positive-sense RNA genomes [26,42,43].

Very few reports have demonstrated the SIE phenomenon at the organism level, for example, in some plant viruses, alphaviruses and flaviviruses in mosquitoes, duck hepatitis B virus, and CSFV in swine [26,42,43,44]. The phenomenon of superinfection, as previously described in CSFV persistently infected pigs, excludes the secondary infection by the highly virulent CSFV strain by the attenuated virus, protecting the animals from the lethal disease in the absence of IFN-α response [26]. Although this phenomenon must be studied in greater depth, the potential of SIE to confer rapid protection to effectively extinguish the circulation of pathogenic strains needs to be thoroughly investigated.

According to the recommendations of *The WOAH Manual for CSF Vaccine Safety Testing in Pregnant Sows*, a live attenuated CSFV vaccine that replicates and is detected in foetuses a week or two before farrowing should not be accepted [11]. Therefore, the next step is the study of the replication kinetics of the FlagT4G vaccine until farrowing.

## 5. Conclusions

The present study demonstrates, for the first time, the high efficacy of a single dose of FlagT4G vaccine in pregnant sows at 44 days of gestation in terms of providing solid protection against highly virulent CSFV transplacental transmission. This constitutes an added value for this vaccine with respect to the existing ones.

## Figures and Tables

**Table 1:** Kinetics of neutralizing antibody response in FlagT4G-vaccinated and non-vaccinated sows.

		FlagT4G Vaccine (Genotype 1.2)			CSFV Margarita Strain (Genotype 1.4)			CSFV Diepholz Strain (Genotype 2.3)			CSFV Alfort/187 Strain (Genotype 1.1)		
	Sow ID	21 dpv	7 dpc	14 dpc	21 dpv	7 dpc	14 dpc	21 dpv	7 dpc	14 dpc	21 dpv	7 dpc	14 dpc
FlagT4G vaccinated	1	1280	2560	1260	40	640	640	320	640	160	160	320	640
	2	320	1280	1280	40	320	640	80	1280	5120	20	80	160
	3 ^†^	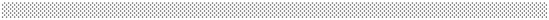											
	4	640	320	1260	40	160	160	160	320	128	40	80	160
Non-vaccinated	5	(−)	(−)	80 *	(−)	(−)	160 *	(−)	(−)	160 *	(−)	(−)	10 *
	6	(−)	(−)	(−)	(−)	(−)	(−)	(−)	(−)	(−)	(−)	(−)	(−)

dpv = day/s post-vaccination; dpc = day(s) post-challenge; 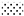 = no sample; (−) = negative sample; ^†^ = sow euthanized at 9 dpv; * = sample taken at 12 dpc.

## Data Availability

The original contributions presented in the study are provided within the manuscript.

## References

[1] Ganges L., Crooke H.R., Bohórquez J.A., Postel A., Sakoda Y., Becher P., Ruggli N. (2020). Classical swine fever virus: The past, present and future. Virus Res..

[2] Tautz N., Tews B.A., Meyers G. (2015). The Molecular Biology of Pestiviruses. Adv. Virus Res..

[3] Rümenapf T., Unger G., Strauss J.H., Thiel H.J. (1993). Processing of the envelope glycoproteins of pestiviruses. J. Virol..

[4] Moennig V., Floegel-Niesmann G., Greiser-Wilke I. (2003). Clinical Signs and Epidemiology of Classical Swine Fever: A Re-view of New Knowledge. Vet. J..

[5] Bohórquez J.A., Muñoz-González S., Pérez-Simó M., Muñoz I., Rosell R., Coronado L., Domingo M., Ganges L. (2020). Foetal immune response activation and high replication rate during generation of classical swine fever congenital infection. Pathogens.

[6] Van Oirschot J., Terpstra C. (1977). A congenital persistent swine fever infection. I. Clinical and virological observations. Vet. Microbiol..

[7] Van Oirschot J. (1979). Experimental production of congenital persistent swine fever infections: II. Effect on functions of the immune system. Vet. Microbiol..

[8] Coronado L., Bohórquez J.A., Muñoz-González S., Perez L.J., Rosell R., Fonseca O., Delgado L., Perera C.L., Frías M.T., Ganges L. (2019). Investigation of chronic and persistent classical swine fever infections under field conditions and their impact on vaccine efficacy. BMC Vet. Res..

[9] Liess B. (1984). Persistent infections of hog cholera: A review. Prev. Vet. Med..

[10] Henke J., Carlson J., Zani L., Leidenberger S., Schwaiger T., Schlottau K., Teifke J.P., Schröder C., Beer M., Blome S. (2018). Protection against transplacental transmission of moderately virulent classical swine fever virus using live marker vaccine “CP7_E2alf”. Vaccine.

[11] World Organisation for Animal Health (WOAH) (2022). Chapter 3.9.3: Classical Swine Fever Virus (infection with Classical Swine Fever Virus). WOAH Terrestrial Manual.

[12] Meyer D., Loeffen W., Postel A., Fritsche S., Becher P. (2018). Reduced specificity of E ^rns^ antibody ELISAs for samples from piglets with maternally derived antibodies induced by vaccination of sows with classical swine fever marker vaccine CP7_E2alf. Transbound. Emerg. Dis..

[13] Suárez-Pedroso M., Sordo-Puga Y., Sosa-Teste I., Rodriguez-Molto M.P., Naranjo-Valdes P., Sardina-Gonzalez T., Santana-Rodriguez E., Montero-Espinosa C., Frias-Laporeaux M.T., Fuentes-Rodriguez Y. (2021). Novel chimeric E2CD154 subunit vaccine is safe and confers long lasting protection against classi-cal swine fever virus. Vet. Immunol. Immunopathol..

[14] Muñoz-González S., Sordo Y., Pérez-Simó M., Suarez M., Canturri A., Rodriguez M.P., Frías-Lepoureau M.T., Domingo M., Estrada M.P., Ganges L. (2018). Corrigendum to “Efficacy of E2 glycoprotein fused to porcine CD154 as a novel chimeric subunit vaccine to prevent classical swine fever virus vertical transmission in pregnant sows”. Vet. Microbiol..

[15] Holinka L., Fernandez-Sainz I., Sanford B., O’Donnell V., Gladue D., Carlson J., Lu Z., Risatti G., Borca M. (2014). Development of an improved live attenuated antigenic marker CSF vaccine strain candidate with an increased genetic stability. Virology.

[16] Bohórquez J.A., Defaus S., Rosell R., Pérez-Simó M., Alberch M., Gladue D.P., Borca M.V., Andreu D., Ganges L. (2021). Development of a dendrimeric peptide-based approach for the differentiation of animals vaccinated with flagt4g against classical swine fever from infected pigs. Viruses.

[17] Bohórquez J.A., Wang M., Díaz I., Alberch M., Pérez-Simó M., Rosell R., Gladue D.P., Borca M.V., Ganges L. (2022). The FlagT4G Vaccine Confers a Strong and Regulated Immunity and Early Virological Protection against Classical Swine Fever. Viruses.

[18] Holinka L.G., O’donnell V., Risatti G.R., Azzinaro P., Arzt J., Stenfeldt C., Velazquez-Salinas L., Carlson J., Gladue D.P., Borca M.V. (2017). Early protection events in swine immunized with an experimental live attenuated classical swine fever marker vaccine, FlagT4G. PLoS ONE.

[19] Vannier P., Plateau E., Tillon J.P. (1981). Congenital tremor in pigs farrowed from sows given hog cholera virus during pregnancy. Am. J. Vet. Res..

[20] Kaden V., Lange E., Steyer H., Lange B., Klopfleisch R., Teifke J., Bruer W. (2008). Classical swine fever virus strain “C” protects the offspring by oral immunisation of pregnant sows. Vet. Microbiol..

[21] Wensvoort G., Terpstra C., Boonstra J., Bloemraad M., Van Zaane D. (1986). Production of monoclonal antibodies against swine fever virus and their use in laboratory diagnosis. Vet. Microbiol..

[22] Reed L.J., Muench H. (1938). A simple method of estimating fifty per cent endpoints. Am. J. Epidemiol..

[23] Terpstra C., Bloemraad M., Gielkens A.L.J. (1984). The neutralizing peroxidase-linked assay for detection of antibody against swine fever virus. Vet. Microbiol..

[24] Terpstra C., Wensvoort G. (1988). The protective value of vaccine-induced neutralising antibody titres in swine fever. Vet. Microbiol..

[25] Hoffmann B., Beer M., Schelp C., Schirrmeier H., Depner K. (2005). Validation of a real-time RT-PCR assay for sensitive and specific detection of classical swine fever. J. Virol. Methods.

[26] Muñoz-González S., Pérez-Simó M., Colom-Cadena A., Cabezón O., Bohórquez J.A., Rosell R., Pérez L.J., Marco I., Lavín S., Domingo M. (2016). Classical swine fever virus vs. Classical swine fever virus: The superinfection exclusion phenome-non in experimentally infected wild boar. PLoS ONE.

[27] Wang M., Bohórquez J.A., Muñoz-González S., Gerber M., Alberch M., Pérez-Simó M., Abad X., Liniger M., Ruggli N., Ganges L. (2022). Removal of the E ^rns^ RNase Activity and of the 3′ Untranslated Region Polyuridine Insertion in a Low-Virulence Classical Swine Fever Virus Triggers a Cytokine Storm and Lethal Disease. J. Virol..

[28] Lowings P., Ibata G., Needham J., Paton D. (1996). Classical swine fever virus diversity and evolution. J. Gen. Virol..

[29] Hall T. (1999). BioEdit: A user-friendly biological sequence alignment editor and analysis program for Windows 95/98/NT. Nucleic Acids.

[30] de Arce H.D., Artursson K., L’Haridon R., Perers A., La Bonnardiere C., Alm G. (1992). A sensitive immunoassay for porcine interferon-α. Vet. Immunol. Immunopathol..

[31] Guzylack-Piriou L., Balmelli C., McCullough K.C., Summerfield A. (2004). Type-A CpG oligonucleotides activate exclusively porcine natural interferon-producing cells to secrete interferon-α, tumour necrosis factor-α and interleukin-12. Immunology.

[32] Kaden V., Lange E., Riebe R., Lange B. (2004). Classical Swine Fever Virus Strain ‘C’. How Long is it Detectable After Oral Vac-cination?. J. Vet. Med. Ser. B.

[33] Summerfield A., Ruggli N. (2015). Immune Responses Against Classical Swine Fever Virus: Between Ignorance and Lunacy. Front. Vet. Sci..

[34] Kaden V., Lange E., Polster U., Klopfleisch R., Teifke J.P. (2004). Studies on the Virulence of Two Field Isolates of the Classical Swine Fever Virus Genotype 2.3 *Rostock.* in Wild Boars of Different Age Groups. J. Vet. Med. Ser. B.

[35] Maciag S., Volpato F., Bombassaro G., Forner R., Oliveira K.P., Bovolato A.L.C., Lopes L., Bastos A.P. (2022). Effects of freezing storage on the stability of maternal cellular and humoral immune components in porcine colostrum. Vet. Immunol. Immunopathol..

[36] Folimonova S.Y. (2012). Superinfection Exclusion Is an Active Virus-Controlled Function That Requires a Specific Viral Protein. J. Virol..

[37] Soller A., Epstein H. (1965). Biochemical and immunological aspects of the exclusion of lambda by superinfection with T4. Virology.

[38] Ramírez S., Pérez-Del-Pulgar S., Carrión J.A., Coto-Llerena M., Mensa L., Dragun J., García-Valdecasas J.C., Navasa M., Forns X. (2010). Hepatitis C virus superinfection of liver grafts: A detailed analysis of early exclusion of non-dominant virus strains. J. Gen. Virol..

[39] Yuan H., Rao J., Zhang J., Ye J., Cao S., Chen H., Song Y. (2024). Japanese encephalitis virus inhibits superinfection of Zika virus in cells by the NS2B protein. J. Virol..

[40] Cwick J.P., Owen J.E., Kochetkova I., Hain K.S., Van Horssen N., Taylor M.P. (2022). Superinfection Exclusion of Alphaherpesviruses Interferes with Virion Trafficking. Microbiol. Spectr..

[41] Burkard T., Proske N., Resner K., Collignon L., Knegendorf L., Friesland M., Verhoye L., Sayed I.M., Brüggemann Y., Nocke M.K. (2022). Viral Interference of Hepatitis C and E Virus Replication in Novel Experimental Co-Infection Systems. Cells.

[42] Newman C.M., Cerutti F., Anderson T.K., Hamer G.L., Walker E.D., Kitron U.D., Ruiz M.O., Brawn J.D., Goldberg T.L. (2011). Culex Flavivirus and West Nile Virus Mosquito Coinfection and Positive Ecological Association in Chicago, United States. Vector-Borne Zoonotic Dis..

[43] Lee Y.-M., Tscherne D.M., Yun S.-I., Frolov I., Rice C.M. (2005). Dual Mechanisms of Pestiviral Superinfection Exclusion at Entry and RNA Replication. J. Virol..

[44] Walters K.-A., Joyce M.A., Addison W.R., Fischer K.P., Tyrrell D.L.J. (2004). Superinfection Exclusion in Duck Hepatitis B Virus Infection Is Mediated by the Large Surface Antigen. J. Virol..

